# Safety and effectiveness of thermal radiofrequency applied to the musculocutaneous nerve for patients with spasticity

**DOI:** 10.3389/fneur.2024.1369947

**Published:** 2024-06-10

**Authors:** Sergio Otero-Villaverde, Jacobo Formigo-Couceiro, Rosa Martin-Mourelle, Antonio Montoto-Marques

**Affiliations:** Physical Medicine and Rehabilitation Department, Complexo Hospitalario Universitario A Coruña, A Coruña, Spain

**Keywords:** radiofrequency, spasticity, musculocutaneous nerve, thermal radiofrequency ablation, disabilility

## Abstract

**Objective:**

Evaluate safety and effectiveness of thermal radiofrequency in the musculocutaneous nerve in patients with focal elbow flexor spasticity.

**Design:**

Ambispective observational follow-up study. Patients with focal spasticity secondary to central nervous system injury with elbow flexor pattern who received thermal radiofrequency treatment in the musculocutaneous nerve between 2021 and 2023 were included.

**Subjects:**

12 patients.

**Methods:**

Ultrasound-guided thermal radiofrequency was applied to the musculocutaneous nerve at 80°C for 90 s. Effectiveness was assessed prior to thermal radiofrequency and at 6 months using scales to measure pain (VAS), spasticity (MAS), disability (DAS), quality of life (SQol-6D), patient-perceived and physician-perceived satisfaction (PIG-C, PGA), and goal attainment (GAS). Elbow joint range of motion was evaluated via goniometry. Safety was evaluated by assessing side effects.

**Results:**

Patients had statistically significant improvements in spasticity (*p* = 0.003), severe elbow flexion (*p* = 0.02), pain (*p* = 0.046), functioning (*p* < 0.05), and spasticity-related quality of life (*p* < 0.05 in three sections). Furthermore, treatment goals were attained. Patient- and physician-perceived clinical improvement was achieved. Regarding side effects, two patients had dysesthesia that was self-limiting, with maximum duration of 1 month.

**Conclusion:**

Thermal radiofrequency in the musculocutaneous nerve can be a safe, effective treatment for patients with severe spasticity with an elbow flexor pattern.

## Introduction

Spasticity is caused by cerebral or spinal central nervous system injury and forms part of upper motor neuron syndrome (1). It entails a very broad and highly variable range of clinical consequences. They can range from nothing of importance or mild or even beneficial effects (2) to severe repercussions for the patient, including pain, joint deformity, difficulty in hygiene, or interference with functioning and basic activities of daily living (3), causing severe problems and affecting quality of life (4, 5) for both the patient and caregivers.

In some patients, focal spasticity may cause joint movement limitation; hinder patient positioning, hygiene, and management by the caregiver; and can even cause pain when it is severe. In select cases, peripheral nerve neurolysis is a treatment option to consider when other more conservative options have failed (6).

Radiofrequency (7, 8) (RF) aims to produce a therapeutic effect in patients using an electrical current. Its use in medicine is not new; indeed, it has been used in highly varied diseases such as cancers or cardiological diseases and especially as a technique for pain relief, forming part of routine clinical practice in pain units, among other indications. Therefore, there is very robust evidence on its safety if used in the appropriate conditions. In regard to the use of RF in patients with spasticity, the literature is scarce and limited. Most publications are on its use in the axial area (9–13) applied to the dorsal and lumbar roots or ganglia or on its use as a means for performing a rhizotomy (14–17). However, regardless of location, the studies are of poor methodological quality. In regard to the use of RF in the peripheral nerve, there is even less scientific support, with just two publications (18, 19) that are described below. Therefore, the use of RF in spasticity is very uncommon nowadays—it has practically fallen into disuse—which is reflected in the age of many of the publications. The current level of evidence that supports its use is very low.

There are only two publications in the literature on performing neurolysis using thermal radiofrequency (TRF), both of which are case reports on a single patient. The first publication is quite old (18), dating to 1987, and the other is recent, from 2023 (19). The results are positive, but they offer a very low level of scientific evidence. No other publications were found on the use of radiofrequency in any of its forms—thermal, pulsed, or cold—on the peripheral nerve for patients with spasticity.

This article aims to evaluate the safety and effectiveness of treatment with TRF applied to the musculocutaneous nerve (MCN) in patients with severe elbow flexor spasticity secondary to central nervous system injury that is refractory to the usual conservative treatment with physical therapy and botulinum toxin type A (BTA).

## Methods

### Participants

This research study was conducted in the Physical Medicine and Rehabilitation Department Outpatient Clinics of the A Coruña University Hospital Complex.

All patients in follow-up with the Rehabilitation Department who met the following inclusion criteria were selected: (1) Signed informed consent form. (2) 18 years of age or older. (3) Diagnosis of spasticity secondary to central nervous system injury. (4) Elbow flexor spasticity pattern (5) Severe elbow flexor spasticity pattern refractory to conservative treatment including physical therapy, orthotics, oral pharmacology, and BTA. (6) Patients have undergone treatment with TRF in our rehabilitation service since its implementation in 2021 to 2023.

All patients with exclusion criteria were not selected: (1) Informed consent form no signed. (2) Age under 18 years. (3) RF treatment applied to patients without central nervous system injury. (4) RF application to any spastic pattern other than the elbow flexor pattern. (5) Any other non-thermal RF modalities. (6) Failure to follow up after RF treatment, for any reason, for at least 6 months.

### Intervention

This is an ambispective observational follow-up study. This study has a retrospective and a prospective part. The retrospective part encompasses the period from January 2021 to September 2022 (seven patients) who had had the technique performed. The prospective part encompasses the period from October 2022 to June 2023 (five patients) who agreed to have the procedure performed. All patients included in this study signed an informed consent form for having the radiofrequency technique performed and for publication of the results.

This study was approved by the Drug Research Ethics Committee of Galicia (DREC-G) with a favorable ruling and registration code 2023/153.

#### Hypothesis

TRF in the MCN is a safe and effective treatment for severe spasticity in patients with an elbow flexor pattern that is refractory to conventional treatments.

#### Primary endpoint

**Figure 1:**
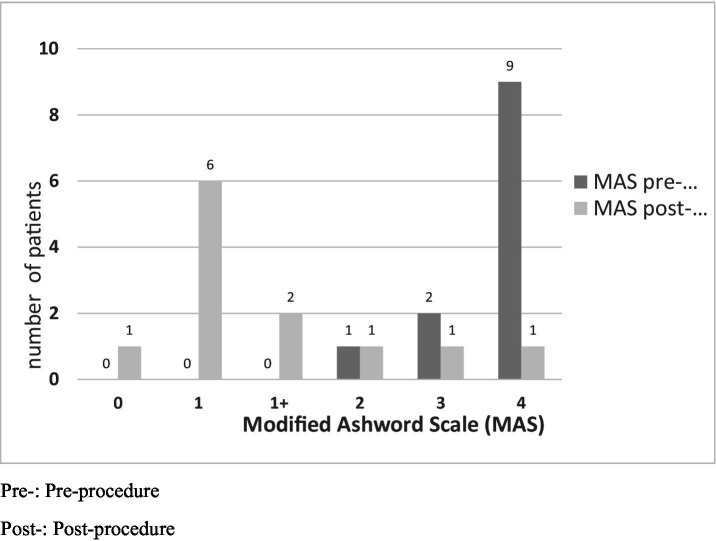
Pre-: Pre-procedure. Post-: Post-procedure.

**Figure 2:**
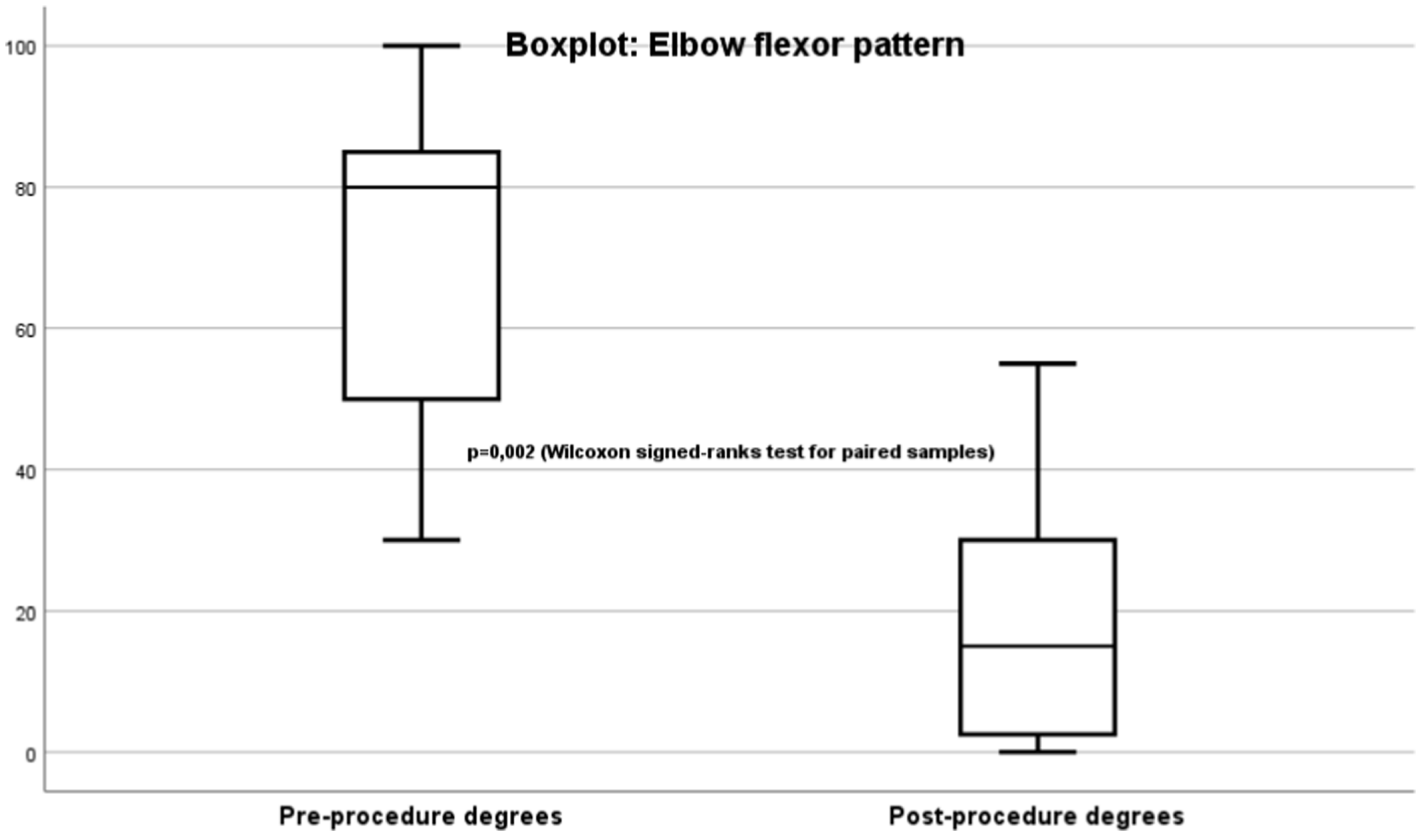
Elbow flexor pattern degrees.

The primary endpoint is spasticity evaluated using the Modified Ashworth Scale (MAS) (20) (Figure 1). Improvement was mainly determined by improvement in the joint range of motion limitation measured in degrees using goniometry (Figure 2).

#### Secondary endpoints

Secondary endpoints were evaluated using the following scales: Visual Analog Scale (VAS) (21), Goal Attainment Scaling (GAS) (22), Physician Global Assessment (PGA) (23), Patient Global Impression of Change (PGI-C) (24), Disability Assessment Scale (DAS) (25), and Spasticity-related Quality Of Life 6-Dimensions (SQoL-6D) (26). Any side effects that occurred were evaluated.

### Duration of radiofrequency effect time: minimum follow-up of 6 months

#### Description of the intervention

**Figure 3:**
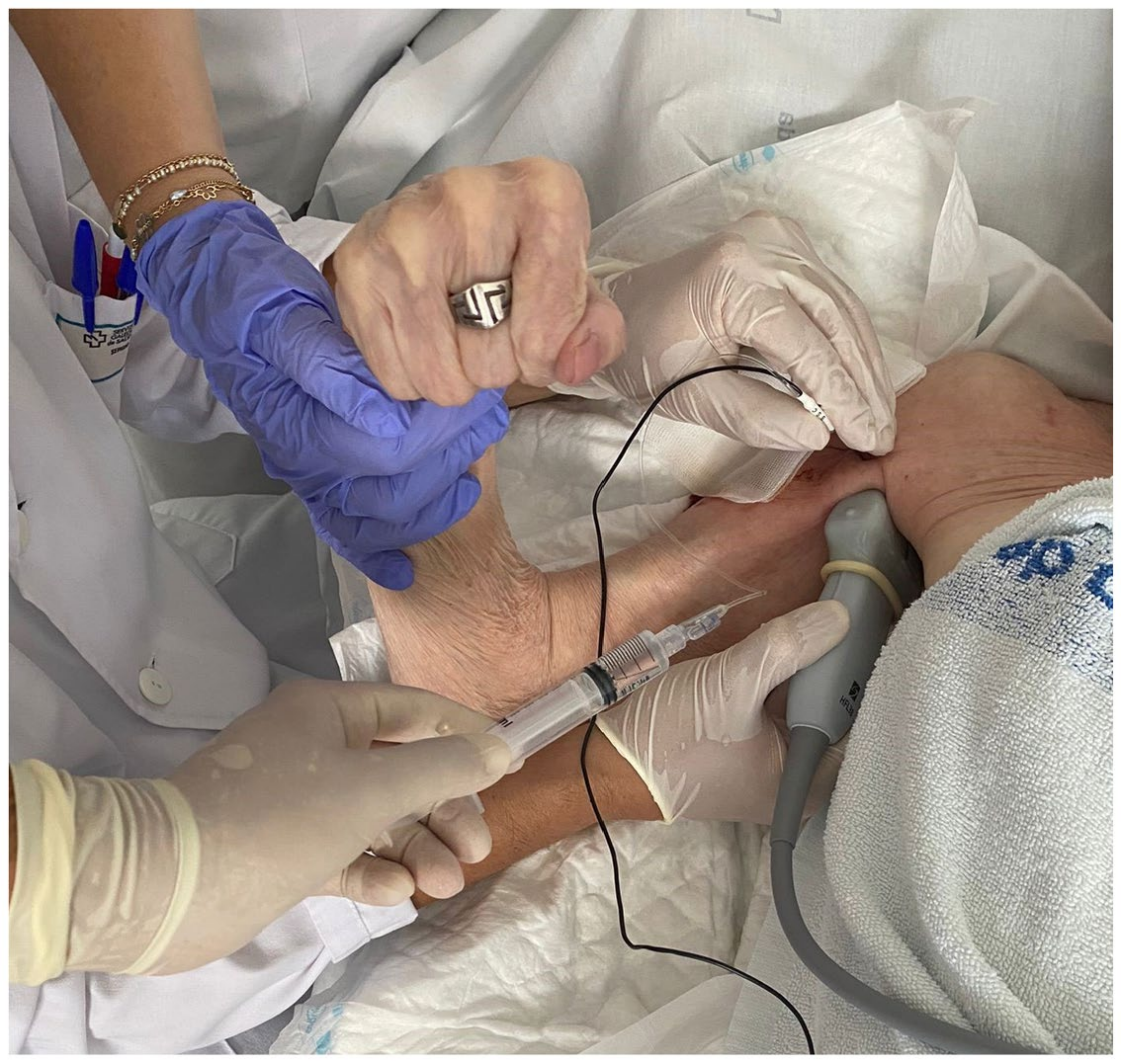
TRF in MCN technique.

**Figure 4:**
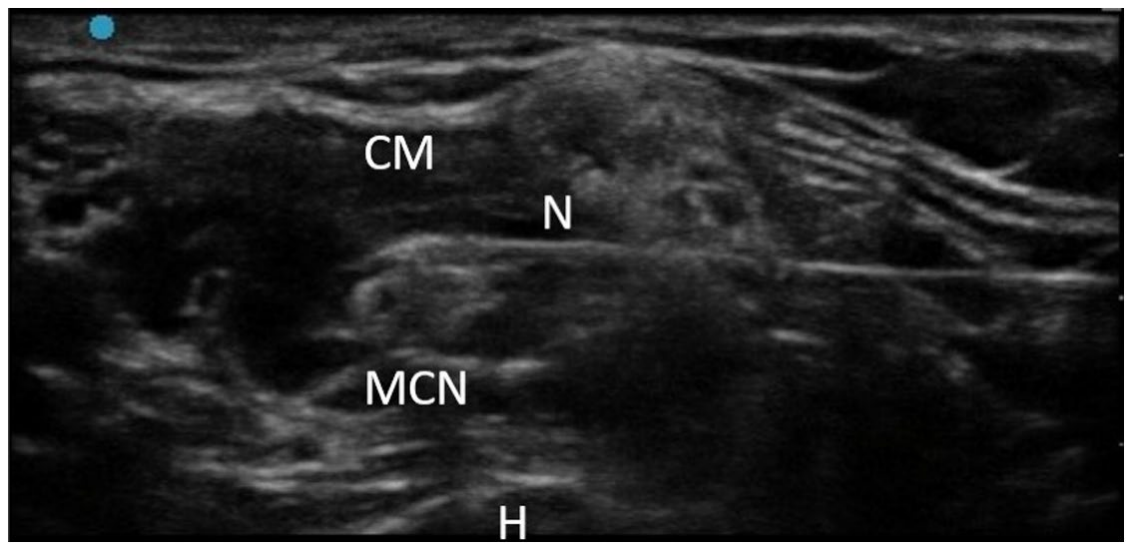
TRF in MCN technique. CM, Coracobrachialis muscle; N, Needle; MCN, Musculocutaneous nerve; H, Humerus.

The intervention involved the use of RFT on the MCN according to the usual protocol performed in the A Coruña University Hospital Physical Medicine and Rehabilitation Department. RFT is the application of an alternating current of low intensity and very high frequency: 500,000 Hz. When the current passes through the tissues, they present a resistance, so part of the current is converted into heat, which is the physical principle that we want to apply by raising the temperature to 80°C, thus causing nerve ablation. The RF equipment was a G4™ RF Generator G4™ Cosman. Marlborough, Massachusetts. To perform the TRF procedure, grounding pads were placed separate from the electrode in the patient’s body. After antiseptic measures in the area to be treated, the MCN was located using ultrasound (Fujifilm Sonosite SII ultrasound machine, 6–13 MHz linear probe; Figure 3). Using an in-plane ultrasound-guided approach, a temperature-controlled, 6-cm, 22G RFT cannula with a 5-mm active tip was introduced toward the MCN (Figure 4). Then, the sensory nerve was stimulated at <0.5 V. It was considered positive if paresthesia occurred in the anatomic location innervated by the MCN. After, motor stimulation at <0.5 V was performed and was considered positive if elbow flexor muscle contraction was observed. With positive nerve stimulation ensuring the appropriate location and distance of the cannula, an MCN block was performed with the injection of 4–6 mL of mepivacaine 2%. The local anesthetic was allowed to take effect for 4 mins and once effective analgesia was reached, the TRF procedure began, reaching a temperature of 80°C for 90 s. Afterwards, the procedure was completed with the injection of one 2-ml vial of betamethasone (Celestone® Cronodose suspension for injection). To summarize, the whole procedure is done at the same time with the same cannula, first the nerve is located with ultrasound, then the cannula is inserted in the direction of the nerve, the stimulation is performed to ensure its correct positioning, the nerve block is performed with local anesthetic so that the procedure is not painful, and then TRF is activated reaching 80°C.

### Statistical analysis

The SPSS Statistics software package was used for the statistical analysis (27). The result of a test was considered significant if the α error was less than or equal to 0.05. Given the sample’s characteristics—especially the small sample size—a non-parametric test was used for comparisons, namely the Wilcoxon signed-ranks test for paired samples.

## Results

Twelve patients with severe focal elbow spasticity refractory to conventional treatment who received TRF of the MCN were included. Of the 12 patients, 10 were men and two were women. The mean age was 58.3 years (range 39–81). The most common neurological disease was stroke, which affected seven patients (four ischemic and three hemorrhagic), followed by three patients with traumatic brain injuries, one patient with cerebral palsy, and one patient with Parkinson-plus syndrome. Of the 12 procedures, four were performed in the right upper limb and eight in the left.

Before undergoing TRF treatment, all patients had had conservative treatment, which at minimum consisted of conventional physical therapy and BTA, without reaching the goals set in regard to the elbow flexor pattern.

Prior to TRF, most patients had severe spasticity with an elbow flexor pattern as defined by the MAS scale (Figure 1). Nine patients had MAS 4/4, two patients had 3/4, and one patient had 2/4. After the procedure, all patients except one had clinical improvement. One patient had MAS 0/4; six had 1/4; two had 1+/4; one had 2/4; one had 3/4; and one did not improve, continuing to score 4/4. These findings were statistically significant (*p* = 0.003). Therefore, it can be concluded that TRF is an effective treatment for spasticity with an elbow flexor pattern.

Most patients had severe elbow flexor pattern (Figure 2) evaluating using goniometry. For the goniometric measurements, a manual goniometer with a fulcrum and scale in degrees was placed on the lateral face of the elbow. Prior to TRF, the patients had a mean non-reducible passive joint stiffness in maximum extension of −70.4° (range: −30° to −100°). After treatment, they had clear clinical improvement, with a mean flexion of −20° (range: 0° to −55°). This finding was also statistically significant (*p* = 0.02).

With respect to pain evaluated using the VAS scale (Table 1), patients also had a statistically significant improvement (*p* = 0.046) after treatment.

**Table 1:** VAS.

VAS	No pain		Mild		Moderate		Severe	
	Pre	Post	Pre	Post	Pre	Post	Pre	Post
*n*	5	7	1	3	4	2	2	0
% (IC 95%)	41.7% (18.0–68.8%)	58.3% (31.2–82.0%)	8.3% (0.9–32.8%)	25.0% (7.6–52.9%)	33.3% (12.5–61.2%)	16.7% (3.6–43.6%)	16.7% (3.6–43.6%)	0.0% (.–.)
*P* ^1^	0.046^*^							

VAS, Visual Analog Scale; Pre, Pre-procedure; Post, Post-procedure. ^1^Significance: Wilcoxon signed-ranks test for paired samples. ^*^Significance (*p* < 0.05).

Functioning was evaluated using the DAS scale (Tables 2, 3). Statistically significant improvement was achieved on its four sections, indicating that patients had improvement in hygiene, dressing, limb position, and pain (*p* < 0.05 for all).

**Table 2:** DAS.

DAS	Pre-procedure hygiene	Post-procedure hygiene	Pre-procedure dressing	Post-procedure dressing
n	12	12	12	12
Mean (SD)^1^	1.5 (0.8)	1.0 (0.6)	2.0 (0.9)	1.3 (0.5)
Median (IQR)^2^	1.5 (1.0–2.0)	1.0 (1.0–1.0)	2.0 (1.0–3.0)	1.0 (1.0–2.0)
*p* ^3^	0.034^*^		0.038^*^	

^1^SD, standard deviation; ^2^IQR, interquartile range; ^3^Significance, Wilcoxon signed-ranks test for paired samples. ^*^Significance (*p* < 0.05).

**Table 3:** DAS.

DAS	Pre-procedure position	Post-procedure position	Pre-procedure pain	Post-procedure pain
*n*	12	12	12	12
Mean (SD)^1^	2.5 (0.5)	1.2 (0.4)	1.3 (1.3)	0.5 (0.7)
Median (IQR)^2^	2.5 (2.0–3.0)	1.0 (1.0–1.0)	1.0 (0.0–2.5)	0.0 (0.0–1.0)
*p* ^3^	0.003^*^		0.047^*^	

^1^SD, standard deviation; ^2^IQR, interquartile range; ^3^Significance, Wilcoxon signed-ranks test for paired samples. ^*^Significance (*p* < 0.05).

The SQoL-6D scale was administered as a tool for evaluating spasticity-related quality of life (Tables 4, 5). Statistically significant improvement was attained in three of its sections: improvement in elbow joint range of movement, improvement in caring for the affected upper limb, and improvement in mobility and/or balance during gait in patients who walk. No significant improvements were observed in the functional use of the upper limb, which stands to reason given that patients have severe spasticity and severe underlying neurological involvement with little or no function or voluntary movement. No statistically significant changes were observed in regard to spasms, given that this was an uncommon symptom in these patients (only present in one patient, with the score decreasing from two before treatment to one after treatment).

**Table 4:** SQoL-6D.

SQoL-6D	Pre-procedure pain	Post-procedure pain	Pre-procedure spasms	Post-procedure spasms	Pre-procedure joint range of movement	Post-procedure joint range of movement
*n*	12	12	12	12	12	12
Mean (SD)^1^	1.1 (1.2)	0.6 (0.7)	0.2 (0.6)	0.1 (0.3)	3.0 (0.9)	1.2 (0.6)
Median (IQR)^2^	1.0 (0.0–2.0)	0.5 (0.0–1.0)	0.0 (0.0–0.0)	0.0 (0.0–0.0)	3.0 (2.0–4.0)	1.0 (1.0–1.5)
*p* ^3^	0.131		0.317		0.003^*^	

^1^SD, standard deviation; ^2^IQR, interquartile range; ^3^Significance, Wilcoxon signed-ranks test for paired samples. ^*^Significance (*p* < 0.05).

**Table 5:** SQoL-6D.

SQoL-6D	Pre-procedure care	Post-procedure care	Pre-procedure use	Post-procedure use	Pre-procedure mobility/balance	Post-procedure mobility/balance
*n*	12	12	12	12	12	12
Mean (SD)^1^	2.3 (0.8)	1.5 (0.7)	4.0 (0.0)	3.9 (0.3)	2.1 (0.8)	1.6 (0.9)
Median (IQR)^2^	2.0 (2.0–3.0)	1.0 (1.0–2.0)	4.0 (4.0–4.0)	4.0 (4.0–4.0)	2.0 (2.0–2.0)	1.0 (1.0–2.0)
*p* ^3^	0.014^*^		0.317		0.014^*^	

^1^SD, standard deviation; ^2^IQR, interquartile range; ^3^Significance, Wilcoxon signed-ranks test for paired samples. ^*^Significance (*p* < 0.05).

Goal attainment was evaluated using the GAS scale. The effect was smaller than expected in two patients (GAS −1), the expected effect was attained in six patients (GAS 0), the effect was greater than expected in three patients (GAS +1), and the effect was much greater than expected in one patient (GAS +2). The mean GAS scale score was 0.3, indicating that the treatment goals were attained.

The PGA scale was used to assess the physician’s impressions of the treatment results, with a mean score of 2.2. The PGI-C scale was used to assess the patient’s subjective improvement, with a mean score of 1.8. Therefore, it can be concluded that patient- and physician-perceived clinical improvement was achieved.

The main aim of this work was not only to evaluate effectiveness, but also to assess safety and monitor side effects. The side effect of dysesthesia occurred in two patients, both of whom reported it in the lateral face of the forearm, but not in the arm or the hand. This area is innervated by the lateral antebrachial cutaneous (LABC) nerve, a purely sensory terminal component of the MCN where it does not have motor function. It was mild in the first patient (VAS 2) and yielded in a self-limiting manner after 2 weeks. It did not require any treatment (no analgesia, which was offered but refused by the patient). The paresthesia was more severe in the other patient (VAS 5) and was controlled with a low dose of pregabalin 75 mg/12 h. It yielded entirely after 1 month, allowing for full suspension of the drug and good progress afterwards. In both cases, the overall improvement was significant and the treatment goals were attained (GAS 0) at 6 months after the procedure. Both continue in follow-up in the Rehabilitation and Physical Medicine Department Outpatient Clinics and have not had recurrence of pain or other complications at 1 year.

## Discussion

The treatment approach to spasticity must be individualized and take into account various factors: etiology, time since onset, prognosis, distribution, location, severity, presence of medical comorbidities, and both the patient’s and caregivers’ treatment goals (28, 29).

The usual treatment options include physical therapy; the use of positioning splints (30, 31); or the use of shock waves, a treatment supported by a growing number of studies (32, 33).

Within pharmacological treatment, there is oral medication (34) such as baclofen, gabapentin, diazepam, or tizanidine or intramuscular drugs such as BTA (35, 36), which is especially indicated in localized focal spasticity in specific muscle groups, allowing for selective action in the target muscle to be treated. The use of intrathecal baclofen pumps can be considered for the treatment of severe generalized spasticity (37). Other techniques were very commonly used in the past, such as denervation with phenol or ethyl alcohol, but are less commonly used at present given the risk of side effects such as paresthesia or dysesthesia described in the literature (38, 39). Techniques such as neuroablation (e.g., DREZotomy) or neuromodulation (e.g., spinal stimulation) are rarely used because they are invasive, carry a higher risk, and their efficacy is poorly established in the literature.

In certain patients with severe spasticity, these treatments may not be enough. Treatment-resistant spastic patterns may persist despite them, causing stiff joint positions that interfere with hygiene and basic activities of daily living such as dressing and eating; hinder walking; and even cause pain that can be severe, with the consequent deterioration in quality of life (40).

TRF treatment in patients with spasticity is a palliative treatment for severe cases that are refractory to habitual conservative management, such as what is described above. Although radiofrequency has been successfully used for years in various diseases, its use in spasticity is novel and at present, there is little scientific support in the literature. It is also not without potential complications, which is why it has been used in so few patients with spasticity to date.

It is recommended to always perform TRF on the MCN with a guidance system, such as ultrasound in this case (41), both for greater precision and safety and to be able to evaluate possible anatomical variations.

The MCN is a mixed nerve (41), but is predominantly motor. It originates in the lateral cord of the brachial plexus. It then enters the coracobrachialis muscle and exits between the brachialis and biceps brachii muscles, providing motor innervation to these three muscles without sensory innervation in the arm. At its entry into the forearm, it is known as the LABC, which is a purely sensory terminal branch innervating the lateral face of the forearm only, without motor innervation of any muscle in this area. Therefore, this nerve is very important in elbow flexor pattern spasticity, given that the biceps brachii and brachialis muscles are responsible for the majority of elbow flexion. This nerve can be used to treat spastic elbow flexor patterns that are resistant to conventional treatment.

An MCN block was performed on the patients in this study. It is always recommended to do so for diagnostic and prognostic purposes. In fact, this technique is a novel manner to differentiate between joint spasticity and ankylosis. In this procedure, the area around the MCN was infiltrated with a local anesthetic. If the block is performed and has a positive effect—that is, the elbow flexion improves significantly—this indicates that there is no ankylosis and therefore the elbow flexion is secondary to spasticity, which is able to be improved with radiofrequency. On the other hand, if the effect of the block is negative—that is, there is no change in elbow flexion—then there is established joint stiffness and neither very high doses of BTA or RF techniques will be effective; surgery would be the only remaining treatment option. Therefore, a nerve block allows for evaluating the potential improvement one can expect to achieve with TRF. This provides a reliable idea of the potential benefit that can be expected with TRF and the patient can then determine whether he or she wants to undergo the procedure or not.

The authors believe that TRF is indicated when there is a positive nerve block of the MCN in patients with severe spasticity refractory to conventional treatments. TRF is a natural next step to a nerve block, as the goal is to prolong the positive effects of acting on a nerve beyond the limited period of time that the effects of a local anesthetic last (6).

In patients with spasticity, if the muscle activity generated by the MCN is clinically detrimental and improves with a nerve block, conventional or thermal radiofrequency would be indicated (6). This causes a nervous system lesion due to elevation of the temperature around the active tip of the cannula to 80°C. This was applied in this protocol for 90 s after using a local anesthetic to prevent pain during the procedure. Afterwards, a corticosteroid injection was given to prolong the local analgesic and anti-inflammatory effect. Thus, the aims of radiofrequency on the MCN are to achieve prolonged improvement in the elbow joint range of motion; improve spasticity; improve pain; improve positioning, including in gait pattern; and ultimately to improve the patient’s and his/her caregivers’ quality of life.

After conducting a literature search, to the best of our knowledge there are just two publications on this technique. Both are case reports on a single patient in which TRF is used for peripheral nerve neurolysis. The first publication is quite old (18), dating to 1987, and describes the simultaneous use of TRF in two peripheral nerves in a single patient: the obturator nerve and the posterior tibial nerve. The other publication is recent (19), from 2023. It is also a single case report in which TRF was used on two motor branches of the femoral nerve and obturator nerve, with positive results. However, these works offer a low level of scientific evidence.

Even regarding RF in any location and of any type, the literature is scarce, heterogeneous, and of low methodological quality. All articles available are either a single case report or a case series. The article with the largest number of patients (16) dates to 1983. It describes 30 patients, most of whom had spastic quadriplegia or paraplegia secondary to traumatic brain injury, who underwent TRF for foraminal rhizotomies. Another article (15) with a similar methodology on 25 patients was published 1 year later, in 1984. There are other articles on pulsed radiofrequency (PRF) used on the dorsal root ganglion (9–13), mostly in pediatric patients (9–12). The publication with the greatest number of patients (11) included 20 children with hip adductor spasticity of heterogeneous etiology; the article offers a low level of scientific evidence as it is a case series. There is another publication on two patients with spinal cord injuries in whom PRF was used on the dorsal root ganglion (13) Lastly, there is a case report on a single patient with traumatic brain injury and severe generalized spasticity of the lower limbs who received TRF in the dorsal root entry area (42). All these studies showed positive results, but offer a low level of scientific evidence.

This article is the most extensive case series on TRF on a peripheral nerve, in this case the MCN, that has been published in the literature. It shows positive results, with improvement of elbow joint range of motion evaluated using goniometry, improvement in severe spasticity according to the MAS scale, and improvement in pain according to the VAS scale. The goals agreed upon with the patient or caregiver were generally achieved, as reflected in the GAS scale; a decline in disability was observed, as assessed by the DAS scale; and there was an improvement in quality of life according to the SQol-6D scale. There was also improvement on the patient’s global impression measured via the PGI-C scale and an improvement in the physician’s global assessment measures via the PGA scale. All of these results were statistically significant.

It should be taken into account that TRF is an ablative treatment in which the nerve is subjected to thermal burning. It is not proposed as first-line treatment, but rather for severe cases in which conservative treatments have failed. It is an interventionist technique and not without potential complications. However, the literature supports its use, with extensive experience in other diseases demonstrating that it is a safe technique when used in the appropriate conditions. In this case series, 16% (two of the 12 patients) had dysesthesia in the lateral antebrachial cutaneous nerve area, but it was mild and self-limiting in time. One case did not require treatment. In the other, the symptoms lasted for 1 month and yielded after administering pregabalin, with no later recurrence and suspension of the drug. Both patients continue in follow-up in the rehabilitation outpatient clinics and have not had any recurrence of pain or other complications after 1 year.

### Limitations

The main limitations of this study are the small sample size and the lack of a control group. It would be desirable to conduct a higher quality, prospective, randomized, placebo-controlled or fenol/alcohol-controlled study in the future. A longer follow-up time would be desirable in order to evaluate the effect over the longer term.

## Conclusion

In light of this study’s results, the authors believe that TRF in the MCN can be a safe, effective technique for treating spasticity in patients with central nervous system injury and a severe elbow flexor pattern. It improves spasticity, elbow joint range of motion, and pain; decreases disability; improves patient- and physician-perceived quality of life; and achieves the treatment goals proposed. What’s more, this technique can be performed in rehabilitation departments outpatient clinics.

This technique can be effective in specific patients and it expands the armamentarium for spasticity treatments. The authors hope this article marks the beginning of an expansion of TRF use for improving the quality of life of patients with spasticity while awaiting future studies of a greater methodological quality that corroborate the suitability of this technique’s widespread use.

## Data availability statement

The original contributions presented in the study are included in the article/supplementary material, further inquiries can be directed to the corresponding author.

## Ethics statement

This study was approved by the Drug Research Ethics Committee of Galicia (DREC-G) with a favorable ruling and registration code 2023/153. The studies were conducted in accordance with the local legislation and institutional requirements. The participants provided their written informed consent to participate in this study. Written informed consent was obtained from the individual(s) for the publication of any potentially identifiable images or data included in this article.

## Author contributions

SO-V: Conceptualization, Data curation, Methodology, Writing – original draft, Writing – review & editing. JF-C: Conceptualization, Supervision, Writing – original draft, Writing – review & editing. RM-M: Writing – original draft, Writing – review & editing. AM-M: Supervision, Writing – original draft, Writing – review & editing.
